# Repeated Cocaine Exposure Attenuates the Desire to Actively Avoid: A Novel Active Avoidance Runway Task

**DOI:** 10.3389/fnbeh.2018.00108

**Published:** 2018-06-01

**Authors:** David Nguyen, Yasika Nesarajah, Suzanne Erb, Rutsuko Ito

**Affiliations:** ^1^Department of Psychology, University of Toronto Scarborough, Toronto, ON, Canada; ^2^Department of Cell and Systems Biology, University of Toronto, Toronto, ON, Canada

**Keywords:** cocaine, sensitization, active avoidance, punishment, incentive motivation, addiction, negative reinforcement, dopamine

## Abstract

Drug addiction is a disorder in which drug seeking persists despite aversive consequences. While it is well documented in animal models of drug sensitization that repeated drug exposure enhances positive incentive motivation for drug and natural reinforcers, its effect on negative incentive motivation, defined here as the motivation to avoid a cued aversive outcome, remains an open question. In the present study, we designed a novel active avoidance (AA) runway paradigm to assess the effects of repeated cocaine exposure on the *motivation* to avoid an aversive outcome. Cocaine and saline pre-exposed rats were first trained to perform a conditioned AA lever press response to prevent the occurrence of foot shock administrations. The rats were subsequently tested in a runway apparatus, wherein they were required to traverse the length of a straight alley maze to reach the lever and emit a conditioned AA response. Run times were measured as an indication of negative incentive motivation. Cocaine pre-exposed rats demonstrated longer latencies to emit the conditioned AA response but showed no differences in latency to initiate runway behavior, nor in their acquisition of the AA response compared to the saline pre-exposed controls. Subsequent testing in an elevated plus maze revealed no differences in the expression of anxiety in cocaine pre-exposed rats compared to saline pre-exposed controls. Our results indicate that prior repeated cocaine exposure attenuated cued negative incentive motivation, which suggests that drug addiction may be attributable to a decrease in motivation to avoid aversive consequences associated with drug use.

## Introduction

Drug addiction is a disorder characterized by compulsive drug-seeking and taking behaviors that persist despite the recurrent experience of negative consequences. Many factors likely contribute to the development of compulsivity, such as the loss of prefrontal cortical-mediated inhibitory control (Goldstein and Volkow, 2011) and aberrant control over drug-seeking behavior by the dorsal striatal-mediated habit system (Everitt and Robbins, 2005). However, it is also possible that compulsive forms of drug-seeking are manifestations of aberrant processing of competing motivational signals: those that simultaneously compel the individual to seek and avoid the drug (Nguyen et al., 2015). It is well-documented that repeated exposure to drugs of abuse such as psychostimulants in preclinical models of drug addiction, causes enduring alterations in mesocorticolimbic DA transmission that are linked to enhanced motivation for rewards and augmented incentive salience of reward-associated stimuli (Kalivas and Stewart, 1991; Robinson and Berridge, 1993; Berridge, 2007). As such, repeated exposure to psychostimulants may induce a shift in the balance of control over behavior by opposing incentive motivational processes (approach and avoidance), and enable approach motivation to gain greater influence over behavior to facilitate compulsive drug seeking in the face of negative consequences.

Furthermore, there is considerable evidence in the preclinical literature that pre-exposure to psychostimulant drugs leads to the potentiation of positive incentive motivation for not only psychostimulants, but also for natural reinforcers (sex, sucrose, food), demonstrating the phenomenon of cross-sensitization (Horger et al., 1990, 1992; Mendrek et al., 1998; Fiorino and Phillips, 1999a,b; Harmer and Phillips, 1999a,b; Wyvell and Berridge, 2001). Conversely, a history of sugar bingeing can lead to increased ethanol intake and a potentiated locomotor response to amphetamine (AMPH; Avena and Hoebel, 2003; Avena et al., 2004). Based on these findings, we recently examined the effect of prior subchronic cocaine exposure on approach-avoidance motivation for a natural sucrose reward paired with foot shock punishment (Nguyen et al., 2015). Using a runway paradigm, cocaine pre-exposed rats were initially trained to traverse a straight alley maze towards a goal compartment containing a well filled with sucrose solution. Once the rats reached stable performance, the delivery of intermittent, inescapable foot shocks was introduced as the rats entered the goal compartment. During this latter phase, cocaine pre-exposed rats exhibited shorter latencies to enter the goal compartment compared to saline pre-exposed controls, despite the aversive consequence of entering the compartment. One way of explaining these findings is to consider that the positive incentive motivation to approach the rewarded compartment was potentiated in cocaine pre-exposed animals, even when aversive consequences were introduced. However, an alternative possibility is that the observed shorter latencies to enter the shock-paired compartment in the cocaine pre-exposed rats were attributable to an attenuation of negative reinforcement, or “negative incentive motivation,” which in this case would apply to the motivation to avoid entering the goal compartment in order to escape the negative foot shock consequences altogether.

In contrast to the abundance of studies examining the effects of repeated drug exposure on positive incentive motivation, the effect of drug sensitization on negative incentive motivation has not been thoroughly investigated. This is likely due to an absence of paradigms that can effectively isolate and quantify a negative incentive motivation value, i.e., how much the animal “desires” to avoid an aversive outcome. Thus, the present study was carried out using a novel paradigm that allowed the assessment of negative incentive motivation in cocaine pre-exposed rats. We first trained rats in an active avoidance (AA) procedure, wherein they were required to lever-press within a given amount of time to avoid the onset of intermittent foot shock administration. Following successful learning of the AA task, the subjects were then tested in a straight alley maze consisting of a start compartment at one end and the conditioned AA lever at the other end. Across trials, runtime was recorded as the rats traversed the straight alley from the start compartment in order to press the lever and avoid foot shock. We report that cocaine relative to saline pre-exposed rats displayed longer latencies to emit the conditioned AA response, indicating reduced motivation to avoid aversive outcomes.

## Materials and Methods

### Subjects

The subjects were male Long Evans rats (Charles River Laboratories, QC, Canada) weighing between 300 g and 400 g at the start of the experiment. All rats were pair-housed and maintained on a 12-h-light/dark cycle (lights on at 7:00 h) at a constant room temperature of 22°C. Water and food were available *ad libitum* throughout the experiment. All behavioral work took place during the light cycle. This study was carried out in accordance with the guidelines of the Canadian Council of Animal Care and the approval of the University of Toronto Animal Care Committee.

### Cocaine Pre-exposure

The cocaine and saline pre-exposure regimen was administered prior to any behavioral testing. This particular regimen was chosen due to its known effectiveness in inducing behavioral sensitization (Churchill et al., 1999). Intraperitoneal (i.p.) injections of cocaine (*n* = 8) or vehicle 0.9% saline (*n* = 6) were administered once daily for seven consecutive days. Prior to injection on days 1 and 7, rats were individually placed in separate transparent plastic chambers (45 cm L × 25 cm W × 20 cm H), wherein locomotor activity was recorded via an Ethovision tracking system (Noldus Information Technology, ON, Canada) for 60 min. After 60 min, rats were removed from the chambers and were administered cocaine (15 mg/kg, i/p.) or saline (i.p.). Rats were immediately returned to the chambers, and locomotor activity was recorded for an additional 60 min. Cocaine (30 mg/kg, i.p.) and saline (i.p.) injections on days 2–6 were administered in a different room, separate from the housing and behavioral testing rooms, and locomotor activity was not recorded on these days. Following the final injection on day 7, rats were left undisturbed in their home cages for 10 days.

### Apparatus

#### Active Avoidance Training

AA training took place in a transparent plexiglass maze chamber consisting of a single enclosed four-walled compartment (50 cm L × 11.5 cm W × 35 cm H) with a removable lid ceiling (Lafayette Instrument Co., IN, USA). The chamber was covered in its entirety with red cellophane wrap to block out external stimuli. Grid flooring consisting of stainless-steel bars spanned the width of the chamber. Each bar was positioned 1.5 cm apart and was connected to an external shock generator (Lafayette Instrument Co., IN, USA). One of the chamber walls consisted of a custom cut wood (varnished) panel. A retractable stainless-steel lever, positioned 6 cm above the floor, and a 28 V stimulus light, positioned directly above the lever, were slotted into the wood panel wall and connected to a PCI interface system (Med Associates, VT, USA). A manually operated white noise generator (TMSOFT, VA, USA) was attached to the external center surface of the lid ceiling.

#### Active Avoidance Runway

AA runway testing took place in a straight alley maze. The maze was identical to the AA training maze chamber in all aspects except for its extended length (200 cm L × 11.5 cm W × 35 cm H) and built-in start compartment (24.5 cm L × 11.5 cm W × 35 cm H) located at the end of the maze opposite to the end containing the retractable lever and stimulus light. A removable stainless-steel guillotine door blocked off the entrance of the start compartment, separating the compartment from the rest of the maze.

### Behavioral Procedures

#### Active Avoidance Training Habituation

Ten days after the final i.p. injection of the pre-exposure injection regimen, rats underwent a single 10-min habituation session in the AA training maze. At session onset, the lever was extended after 5 s and remained extended until a lever press response was emitted. Upon lever pressing, the lever was retracted for 5 s, after which it was again extended. This process was repeated for each lever press response until the session ended.

#### Active Avoidance Training

**Figure 1:**
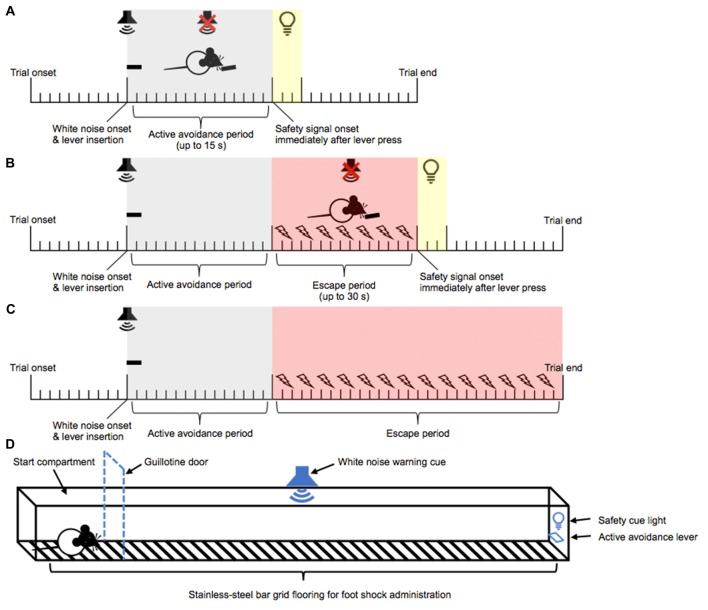
Active avoidance (AA) runway task. Training phase: a continuous white noise and a lever were presented 10 s after trial onset. A lever press within 15 s of white noise onset resulted in the immediate termination of white noise, retraction of the lever, and the onset of a 3 s safety signal light and 15 s no event period. This was classified as an *AA* response **(A)**. Failure to lever press within 15 s of white noise onset triggered the onset of intermittent foot shock. A lever press within 30 s of foot shock onset resulted in the immediate termination of white noise and foot shock, retraction of the lever, and the onset of a 3 s safety signal light and 15 s no event period. This was classified as an *escape* response **(B)**. Failure to lever press within 30 s of foot shock onset resulted in the termination of white noise and foot shock, retraction of the lever, and the immediate onset of a new trial **(C)**. Test phase: following the training and re-training phases, negative incentive motivation was assessed in a runway task. Test trials began in the start compartment (indicated by the dashed line). The white noise warning cue and the lever were presented 10 s after trial onset. Five seconds later, the guillotine door was lifted, allowing access to the straight alley and AA lever **(D)**. The rats then had 15 s within which they could respond to actively avoid oncoming shocks, or a further 30 s in which they could escape the shocks.

Rats were trained over 10 daily trials to “escape” and eventually “avoid” intermittent mild foot shocks. Each training session began with the rat being placed into the center of the maze apparatus. After 10 s of trial onset, rats were presented with a lever, coupled with the presentation of a continuous white noise. A lever press emitted within 15 s of lever and white noise presentation was classified as an “AA” response, and resulted in the termination of the white noise, retraction of the lever, and the onset of a 15 s safety period signaled by a 3 s cue light. A new trial began upon the completion of the safety period. If the rat failed to lever press within the 15 s period, intermittent mild foot shocks (0.27 mA, 0.5 s every 1 s) were initiated. A single lever press at any point after the initiation of the foot shocks was classified as an “escape” response and resulted in the termination of the foot shocks and white noise, retraction of the lever, and a 15 s safety period signaled by a 3 s cue light. A new trial began at the end of the 15 s safety period. A failure to respond on the lever within 45 s of lever and white noise presentation resulted in termination of foot shock and white noise, retraction of the lever, and the immediate onset of a new trial. Daily training continued until animals achieved >80% AA responses (as opposed to escape responses) for two consecutive days (see Figures 1A–C for a timeline of events during AA training).

#### Active Avoidance Runway Habituation and Re-training

Following acquisition of the AA response, the rats were habituated and re-trained on the AA task in the straight alley maze apparatus to reestablish a stable baseline AA performance in a new context. Habituation to the straight alley maze apparatus was carried out in a single session with the guillotine door removed, which allowed access to the entire apparatus. Identical procedures described for the AA training maze apparatus habituation session were used. Rats then underwent seven daily training sessions (10 trials/session), with access to the entire straight alley maze apparatus, following identical procedures described for the previous AA training. In this apparatus, failure to perform the lever press response during the AA period within 15 s of the warning cue onset resulted in foot shocks that were administered from the stainless-steel bar grid flooring along the entire length of the straight alley maze apparatus. Training continued until re-acquisition of the AA response was achieved (>80% AA responses for two consecutive days).

#### Active Avoidance Runway Test

Following re-acquisition of the AA response, rats were tested for negative incentive motivation in five daily AA runway test sessions (see Figure 1D for a schematic of the straight alley maze). Each test trial began with the rat being placed in the start compartment with the entrance to the straight alleyway maze blocked by the guillotine door. The lever and white noise were presented after 10 s of trial onset. Following an additional 5 s, the guillotine door was lifted allowing access into the straight alleyway maze. Latency to leave the start compartment and latency to respond on the lever upon leaving the start compartment were recorded as “start latency” and “goal latency,” respectively. Responding on the lever within 15 s of the guillotine door lifting resulted in the termination of the white noise, retraction of the lever, and a 15 s safety period signaled by a 3 s cue light. The rat was then removed from the apparatus and placed back into the start compartment, initiating the next trial. However, if a lever press response was not emitted within 15 s of the guillotine door lifting, then mild foot shocks were initiated (0.27 mA, 0.5 s every 1 s). A single lever press at any point after the initiation of the foot shocks resulted in the termination of the foot shocks and white noise, retraction of the lever and a 15 s safety period signaled by a 3 s cue light. A new trial began at the end of the 15 s safety period in the start compartment. Furthermore, failure to respond on the lever within 30 s of foot shock onset resulted in the termination of foot shock and white noise, and the rat was immediately removed from the apparatus and placed into the start compartment to initiate a new trial. Each session ended upon the completion of five trials that resulted in the emission of an AA response. A session was typically completed within a range of 5–8 trials across the five test days.

#### Warning Cue Test

A single test session consisting of 10 trials was subsequently conducted under extinction conditions (no foot shock). This test served to confirm that runway behavior and lever press responses were under the control of the white noise cue, and hence the rats’ motivation to avoid oncoming foot shocks, rather than any other factors such as the presentation of the lever itself or lever pressing for the presentation of the 3 s light stimulus (safety signal). Each trial began with the rat being placed in the start compartment. In 5 of the 10 trials, the lever and white noise were presented after 10 s of trial onset. The guillotine door was lifted after an additional 5 s, allowing access to the straight alleyway. The emission of a lever press at any point resulted in the termination of the white noise, retraction of the lever, and a 15 s period with no scheduled event signaled by a 3 s cue light. The rat was then removed from the apparatus and placed into the start compartment to initiate the next trial. The other five trials were performed using the same procedures with the exception that the white noise warning cue was not presented. Lever pressing at any point after the guillotine door was lifted resulted in retraction of the lever and a 15 s period, with no scheduled event, that was signaled by a 3 s cue light. The order in which white noise-cued and non-cued trials were presented was randomized. Furthermore, if a lever press was not emitted within 45 s of the guillotine door lifting, the rat was immediately removed from the apparatus and placed into the start compartment, and a new trial was initiated.

#### Safety Signal Test

Finally, rats underwent one more test session in which they were subjected to a sequence of three events under extinction conditions in order to assess whether the safety signal had developed instrumental reinforcing properties: (1) the AA lever and safety signal were presented simultaneously for a period of 15 s; (2) the lever was retracted and safety signal switched off for 5 s; and (3) the lever was presented again for a further 15 s in the absence of the safety signal. This sequence was repeated four more times, with an inter-trial interval of 5 s. The number of lever presses emitted during the safety signal on and off phases was recorded.

#### Elevated Plus Maze Test

After completion of the AA runway test, rats were tested for anxiety in the elevated plus maze (EPM). The plus maze apparatus was located in a brightly lit room and elevated 43.2 cm from the ground and consisted of a center area (10 cm L × 10 cm W) radiating four arms (each 43.2 cm L × 10.2 cm W) forming a plus shape. Two arms located directly across from one another were enclosed by high walls (“closed,” 24.8 cm H), and the other two arms were not enclosed (“open”). Rats were tested in a single trial, which began with the rat placed in the center of the maze with the rat’s head orientated towards an open arm. The rat was given free exploration of the entire apparatus for 10 min.

#### Locomotor Challenge Test

After completion of all behavioral assays, rats were tested for locomotor sensitization in response to a low dose cocaine challenge (10 mg/kg, i.p.). Rats were placed into separate transparent plastic chambers (45 cm L × 25 cm W × 20 cm H), wherein baseline locomotor activity was established in a 1 h session recorded with an Ethovision tracking system (Noldus Information Technology, ON, Canada). All rats were then removed from the recording chambers, administered cocaine (10 mg/kg, i.p.), and immediately returned to their recording chambers where locomotor activity was recorded for 1 h.

### Data Analysis

All data analyses were performed with SPSS statistical package version 23.0 (IBM, ON, Canada). AA training and re-training data were analyzed using repeated-measures multivariate analysis of variance (MANOVA). Response type, defined as the number of “escape” and “AA” responses emitted on each training day, was the within-subjects factor and Drug pre-exposure (cocaine or saline) was the between-subjects factor. For the AA runway test data, two-way repeated-measures analysis of variance (ANOVA) was used to compare start and goal latencies of trials wherein an AA response was emitted, across test days, with Test day as the within-subjects variable and Drug pre-exposure as the between-subjects variable. The same analysis was applied to the ratio of AA to non-AA responses that were emitted on each of the five test days. For the AA cue confirmation tests, two-way repeated measures ANOVA was used to compare the number of lever presses or latencies during different trial or event types, with Trial type as the within-subjects variable and Drug pre-exposure as the between-subjects variable. Trials wherein a lever press was not emitted were scored with a goal latency of 45 s. For the EPM test, two-way repeated measures ANOVA was used to compare time spent in the open and enclosed arms, with Arm type as the within-subjects factor and Drug pre-exposure as the between-subjects factor. Locomotor sensitization was assessed at two separate time points using two-way repeated measures ANOVA. First, for the locomotor tests that took place on days 1 and 7 of the drug pre-exposure regimen, total distance traveled post injection was compared between injection day 1 and injection day 7, with Injection day as the within-subjects variable and Drug treatment as the between-subjects variable. Second, for the locomotor challenge test, total distance traveled was compared between pre- and post-challenge tests, with Test as the within-subjects variable and Drug pre-exposure as the between-subjects variable. All significant interactions were further analyzed with multiple pairwise comparisons, which were subjected to a Bonferroni correction.

## Results

### Active Avoidance Training and Re-training

**Figure 2:**
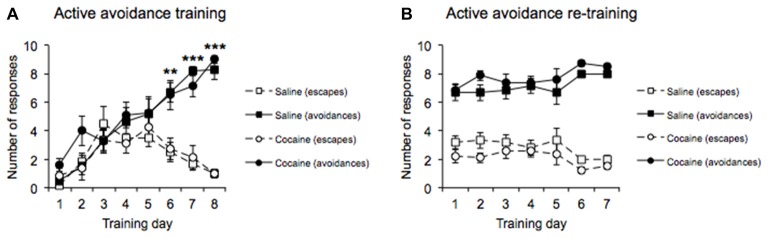
AA acquisition training data across 8 days **(A)** revealed increasing conditioned AA responses and decreasing escape responses as training progressed with no differences between drug pre-exposure conditions. AA re-training data across 7 days **(B)** in the straight alley maze configuration revealed a significantly greater number of conditioned AA responses than escape responses with no differences between drug pre-exposure conditions (±SEM, **p < 0.01, ***p < 0.0001).

MANOVA performed on the AA acquisition data (Figure 2A) revealed a significant main effect of Response type (*F*_(1,12)_ = 30.376, *p* < 0.0001), with the data indicating that more AA responses than escape responses were emitted overall during the AA training phase. A significant main effect of Training day was also revealed (*F*_(7,84)_ = 36.829, *p* < 0.0001), with the data indicating that the overall number of responses emitted increased as training progressed. In addition, there was a significant interaction between Response type and Training day (*F*_(7,84)_ = 11.662, *p* < 0.0001). Subsequent pairwise comparisons revealed that the number of AA responses emitted within each session increased, while the number of escape responses emitted decreased over the course of the 8 days of training (Days 1–5: no difference in active vs. escape responses; all *p*’s > 0.1, Day 6–8: significant difference between active vs. escape responses, Day 6 (*p* < 0.01), Day 7 (*p* < 0.0001), Day 8 (*p* < 0.0001). Crucially, both cocaine and saline pre-exposed groups successfully acquired AA responses (Response type × Drug pre-exposure, *F*_(1,12)_ = 0.196, *p* = 0.666, Training day × Drug pre-exposure, *F*_(7,84)_ = 1.358, *p* = 0.250, Response type × Training day × Drug pre-exposure *F*_(7,84)_ = 0.654, *p* = 0.658).

Following the successful acquisition of the AA lever response, all rats were transferred to training in the runway apparatus, in which they were required to run down a straight alley to emit the AA response. MANOVA performed on the AA re-training data (Figure 2B) revealed a significant main effect of Response type (*F*_(1,12)_ = 139.681, *p* < 0.0001), with the data indicating that AA responses were greater than escape responses overall during the re-training phase. In addition, a main effect of Re-training day was not found (*F*_(6,72)_ = 1.883, *p* = 0.095), indicating that the overall number of responses emitted did not differ across days, and there was no significant difference in AA performance between the cocaine and saline pre-exposed groups (Response type × Drug pre-exposure, *F*_(1,12)_ = 2.565, *p* = 0.135, Re-training day × Drug pre-exposure, *F*_(6,72)_ = 0.871, *p* = 0.521, Response type × Re-training day × Drug pre-exposure, *F*_(6,72)_ = 0.295, *p* = 0.899; Figure 2B).

### Active Avoidance Runway Test

**Figure 3:**
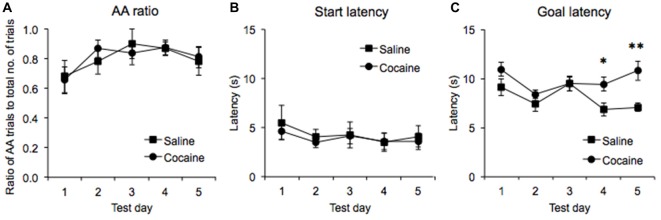
AA runway test data across five test days expressed as the ratio of successful AA responses to the total number of trials in each session **(A)**, the latency to leave the start compartment upon the guillotine door lifting (start latency, **B**), and the latency to lever press upon leaving the start compartment (goal latency, **C**) in saline- and cocaine pre-exposed rats (± SEM, *p < 0.05, **p < 0.01).

Once rats achieved stable running performance in the runway, they were subjected to five daily test sessions in which they were required to emit a minimum of five AA trials. It was found that the ratio of trials in which AA was successfully emitted, to those in which the animals failed to emit an AA response increased significantly across the five test days (main effect of Training Day: *F*_(4,48)_ = 3.43, *p* < 0.04), such that the animals were performing the AA response on 81 ± 0.07 (SEM)% of trials in the cocaine pre-exposed group and on 80 ± 0.06 (SEM)% of the trials in the saline pre-exposed group by Day 5 (Figure 3A). ANOVA confirmed that there were no significant differences in the ratio of AA responses emitted in the cocaine- and saline-pre-exposed groups (no effect of Drug Pre-exposure *F*_(1,12)_ = 0.008, *p* = 0.932, no Test Day × Drug Pre-exposure interaction, *F*_(4,48)_ = 0.405, *p* = 0.706).

ANOVA performed on the start latencies generated in the five AA trials revealed no significant main effect of Test day (*F*_(4,48)_ = 1.473, *p* = 0.225) or interaction between Drug pre-exposure and Test day (*F*_(4,48)_ = 0.141, *p* = 0.966), indicating that start latencies were stable across test sessions and were unaffected by cocaine pre-exposure (Figure 3B).

In contrast, ANOVA performed on goal latencies generated in the five AA trials revealed a significant main effect of Test day (*F*_(4,48)_ = 4.24, *p* < 0.01), with the data suggesting that run times decreased overall as testing progressed. A significant interaction between Test day and Drug pre-exposure was also found (*F*_(4,28)_ = 2.637, *p* < 0.05), and subsequent pairwise comparisons revealed that the goal latencies of the cocaine and saline pre-exposed rats were significantly different on test days 4 (*p* < 0.03) and 5 (*p* < 0.01) but not on test days 1, 2 and 3 (all *p*’s > 0.13) Furthermore, a significant between-subjects effect was found (*F*_(1,12)_ = 6.765, *p* < 0.03), indicating that cocaine pre-exposed rats displayed higher goal latencies overall across the five test days (Figure 3C).

### Warning Cue Test

**Figure 4:**
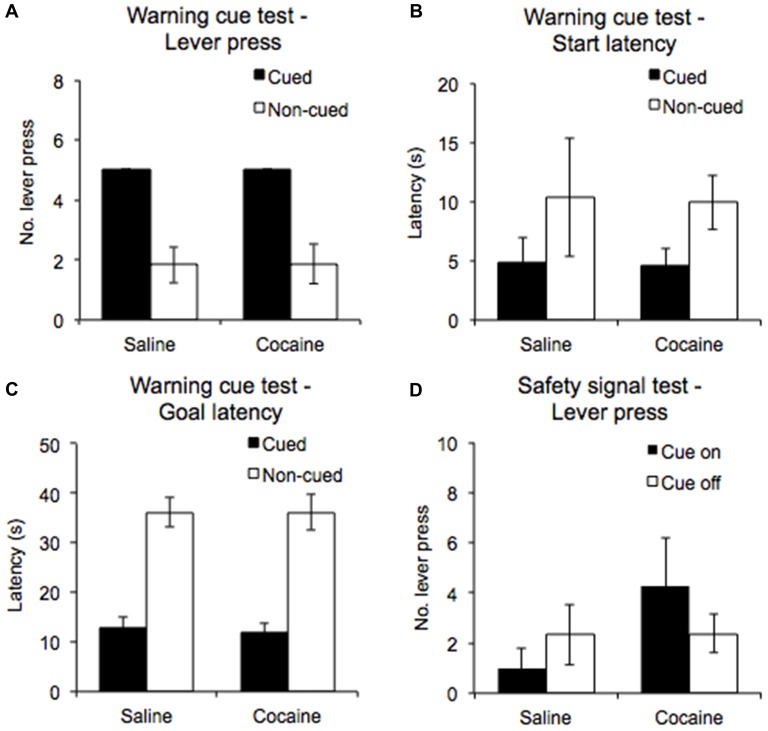
A warning cue test session was conducted to compare the number of lever presses emitted **(A)**, the start latencies **(B)** and goal latencies **(C)** during white noise-cued and non-cued trials in saline- and cocaine- pre-exposed rats. A safety cue test was also conducted, in which the number of lever presses emitted during periods in which the safety signal was on and off was compared **(D)** (±SEM).

During the warning cue test, rats were presented with five cued trials and five non-cued trials in a randomized order to establish the importance of the warning cue in eliciting and sustaining the AA response. Even though the test was performed under extinction conditions, all rats continued to emit a lever press response in the cued trials (100%), while emitting significantly fewer lever press responses in the non-cued trials (see Figure 4A, significant main effect of Trial type: *F*_(1,12)_ = 45.61, *p* < 0.0001). Furthermore, there were no significant differences in the performance of the cue confirmation test between the saline, and cocaine pre-exposure groups (Drug pre-exposure *F*_(1,12)_ = 0.002, *p* = 0.97, Drug pre-exposure × Trial type *F*_(1,12)_ = 0.002, *p* = 0.97).

ANOVA performed on start latencies revealed a significant main effect of Trial type (*F*_(1,12)_ = 11.519, *p* < 0.01), but no significant interaction with Drug pre-exposure (*F*_(1,12)_ = 0.004, *p* = 0.952). The data shown in Figure 4B indicate that start latencies were significantly lower overall for cued trials compared to non-cued trials. For goal latencies, ANOVA revealed a significant main effect of Trial type (*F*_(1,12)_ = 137.3, *p* < 0.0001), with the data indicating that latencies were lower for cued trials compared to non-cued trials. An interaction with Drug pre-exposure was not found (*F*_(1,12)_ = 0.029, *p* = 0.868; Figure 4C).

### Safety Signal Test

The safety cue test was conducted to examine the instrumental reinforcing properties of the safety signal. ANOVA performed on the numbers of lever presses emitted during the safety cue on and off periods (Figure 4D) revealed no significant main effect of Cue (*F*_(1,12)_ = 0.07, *p* = 0.80) nor Drug pre-exposure (*F*_(1,12)_ = 1.03, *p* = 0.33), and no significant interaction between them (*F*_(1,12)_ = 2.32, *p* = 0.15). Thus, the safety signal in and of itself did not elicit/support AA lever pressing.

### EPM Test

**Figure 5:**
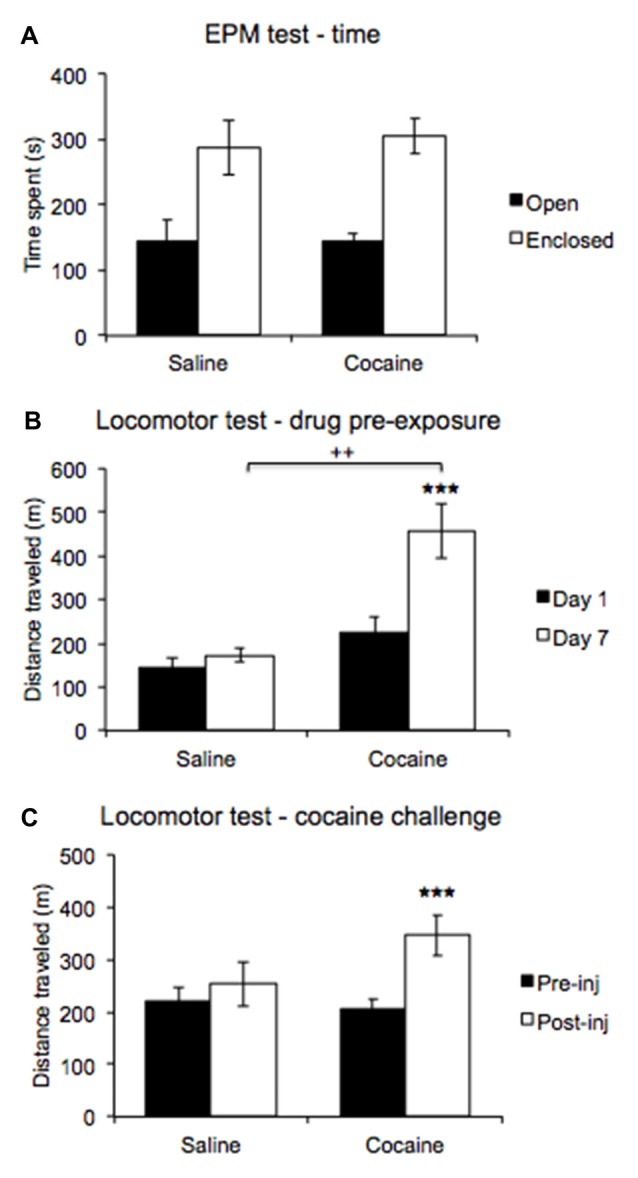
An elevated plus maze (EPM) test compared time spent in open and enclosed arms **(A)** revealing no differences in anxiety expression between pre-exposure conditions. A locomotor test comparing post injection activity on days 1 and 7 of the drug pre-exposure regimen **(B)** revealed increased post-injection activity on day 7 in cocaine treated rats. A locomotor test comparing activity between pre- and post-challenge injection **(C)** revealed a heightened locomotor response in cocaine pre-exposed rats (±SEM, ^++^*p* < 0.01, ***p < 0.000).

ANOVA revealed a significant main effect of Arm type (*F*_(1,11)_ = 18.011, *p* < 0.01), and the data indicate that rats preferred the enclosed to the open arms overall. An interaction with Drug pre-exposure was not significant (*F*_(1,11)_ = 0.048, *p* = 0.83; Figure 5A).

### Locomotor Sensitization

ANOVA comparing post-injection locomotor activity recorded on days 1 and 7 of the drug pre-exposure regimen revealed a significant main effect of Day (*F*_(1,11)_ = 22.877, *p* < 0.001), with the data indicating higher activity overall on injection day 7. A significant interaction with Drug treatment was also found (*F*_(1,11)_ = 13.117, *p* < 0.01). *Post hoc* pairwise comparisons revealed no significant difference in post-injection locomotor activity between cocaine- and saline-treated rats on injection day 1 (*p* = 0.14), but post-injection activity significantly differed between cocaine-treated and saline-treated rats on injection day 7 (*p* < 0.01). Moreover, the data revealed that cocaine-treated rats displayed higher activity post injection on day 7 than on day 1 (*p* < 0.0001), while saline-treated rats did not (*p* = 0.48). Furthermore, a significant between-subjects effect was found (*F*_(1,11)_ = 8.468, *p* < 0.02), with the data indicating that cocaine-treated rats showed higher post-injection activity overall compared to saline-treated rats (Figure 5B).

ANOVA comparing pre- and post-injection activity in the locomotor challenge test conducted upon completion of all behavioral testing revealed a significant main effect of Test (*F*_(1,11)_ = 18.793, *p* < 0.001), with the data indicating higher locomotor activity overall post-cocaine challenge injection. A significant interaction with Drug pre-exposure was also found (*F*_(1,11)_ = 7.081, *p* < 0.03), and *post hoc* pairwise comparisons revealed that cocaine and saline pre-exposed rats did not significantly differ in locomotor activity before (*p* = 0.663) or after the cocaine challenge injection (*p* = 0.143). However, the data indicate that cocaine pre-exposed rats displayed higher locomotor activity post-cocaine challenge injection compared to pre-cocaine challenge injection (*p* < 0.0001), while saline pre-exposed rats did not (*p* = 0.309; Figure 5C).

## Discussion

The present study utilized a novel AA runway task to assess negative incentive motivation, the desire to avoid an aversive outcome, in cocaine pre-exposed rats. We found that cocaine relative to saline pre-exposed rats demonstrated longer run times to a lever located at the opposite end of the straight alley maze, which upon pressing, resulted in the avoidance of foot shock onset. On the other hand, cocaine and saline pre-exposed rats showed no differences in the acquisition of the conditioned AA response. Finally, cocaine pre-exposed rats exhibited heightened locomotor activity in response to a cocaine challenge injection, a response reflecting the expression of cocaine-induced behavioral sensitization and linked to enduring neuroadaptations in dopaminergic systems (Kalivas and Stewart, 1991; Robinson and Berridge, 2000). Our findings reveal that a history of cocaine pre-exposure attenuates negative incentive motivation, which may occur in parallel with enhancements in positive incentive motivation that have been shown by previous studies on animal models of drug sensitization (Lett, 1989; Horger et al., 1990, 1992; Mendrek et al., 1998; Deroche et al., 1999; Fiorino and Phillips, 1999a,b; Harmer and Phillips, 1999a; Wyvell and Berridge, 2001). We propose that this bidirectional change in incentive motivational processing may contribute to the development of compulsive drug seeking behaviors in addicted individuals.

### Repeated Cocaine Exposure and Negative Incentive Motivation

The current study presents novel evidence that repeated exposure to cocaine leads to a *decrease* in the motivation to avoid aversive events (i.e., negative incentive motivation), as indicated by the slower run times of the cocaine relative to saline pre-exposed rats to reach and respond on the conditioned AA lever in the straight alley maze. Interestingly, evidence of a significant attenuation of negative incentive motivation in the cocaine pre-exposed group emerged only when the runway AA performance had become more established, and after the rats had acclimatized to the new procedures introduced in the runway test phase (i.e., by Days 4 and 5). We suggest that repeated subchronic exposure to cocaine led to a lower incentive value being ascribed to the warning cue and/or the foot shock avoidance outcome, thereby decreasing the degree to which shock avoidance was “wanted,” in these rats.

We also propose that the observed attenuation in negative incentive motivation is likely to co-occur with states of heightened positive incentive motivation for both drugs and natural rewards, in accord with the incentive sensitization theory (Robinson and Berridge, 1993, 2000). Indeed, psychostimulant-sensitized animals have been shown to display enhanced acquisition of drug self-administration (Horger et al., 1990, 1992) and drug conditioned place preference (Lett, 1989), increased break points for responding for AMPH in progressive ratio schedules of reinforcement (Mendrek et al., 1998), and enhanced motivation to run in a subsequent test of cocaine seeking in a runway apparatus (Deroche et al., 1999). Similarly, repeated exposure to AMPH has been shown to enhance conditioning to cues associated with sucrose reward (Harmer and Phillips, 1999a; Ito and Canseliet, 2010), and potentiate the Pavlovian to instrumental transfer of a cue associated with sucrose availability (Wyvell and Berridge, 2001).

Additionally, the results of the present study help explain a finding in a previous study from our laboratory, in which we utilized a variation of the runway paradigm and demonstrated that cocaine pre-exposed rats were quicker to enter a goal compartment in which they received both sucrose reward and foot shock punishment (Nguyen et al., 2015). The results suggested that prior cocaine exposure led to a reduction in motivational conflict when approach and avoidance motivations were simultaneously induced (the presence of shock in the goal compartment did not slow down their run times). The results also suggested that the motivational conflict was attributable to drug-induced alterations in basic motivational processes of reward and aversion, processes that may underlie addicts’ tendency to compulsively seek drugs despite the threat of negative consequences. However, it was not possible, based on our previous work, to determine whether the reduction in motivational conflict was due to an increase in positive incentive motivation, a decrease in negative incentive motivation, or a combination of both. The results of the present study provide support for the last possibility, that the reduction in motivational conflict was at least partially attributable to a cocaine pre-exposure-induced attenuation in negative incentive motivation, thereby allowing appetitive incentive motivation to assume greater control over runway behavior.

### Measuring Negative Incentive Motivation: A Novel Runway Paradigm

In the present study, we sought to design a behavioral paradigm that would allow us to measure the motivation to run to avoid an oncoming aversive event. AA paradigms typically use operant chambers, wherein the animal lever presses to avoid cued foot shock onset, or shuttle boxes wherein the animal shuffles across a hurdle separating two ends of a box apparatus to avoid cued foot shock onset (Oleson and Cheer, 2013; Ilango et al., 2014). While studies using these paradigms provide insight into the effects of drug exposure on the acquisition and retention of conditioned AA, the effects of drug exposure on the degree to which the animal *desires* to avoid the aversive event is difficult to assess. For instance, in a free operant AA paradigm, any drug-induced alterations in the AA performance may reflect a change in the animal’s ability to perform the conditioned AA response rather than a change in the animal’s motivation to perform the response. We believe that our AA runway paradigm provides a novel, additional method for quantifying the value of “negative incentive motivation” using run time as a dependent variable that is separable from, and precedes the AA response, much in the same manner as running speed in a runway paradigm being used to measure positive incentive motivation. The latency to traverse the alleyway to the goal compartment is considered to be a measure of the animal’s motivation to attain the reward and, by extension, of the reinforcer’s incentive value (Crespi, 1942; Ettenberg and Geist, 1993; Geist and Ettenberg, 1997; Deroche et al., 1999; Nguyen et al., 2015). In the present study, the latency to run to and respond on the AA lever was measured to provide an index of the animal’s motivation to avoid the cued foot shock event. Importantly, our cocaine and saline pre-exposed rats showed equivalent performance in both the acquisition and re-training phases of the AA lever press response, which enabled us to rule out any potential effects of cocaine pre-exposure on the ability to learn and perform the conditioned AA lever press response. Our results also indicated that anxiety and baseline locomotor activity were unaffected by cocaine pre-exposure. As such, we were able to ascertain that the slower run times exhibited by the cocaine pre-exposed rats were most likely the result of attenuated negative incentive motivation.

Further tests were also conducted to assess whether the animals’ running behavior was governed by the desire to alleviate the delivery of an aversive event (negative reinforcement), or the securement of safety (positive reinforcement). This was important to disentangle, as cues which signal the prospect of safety (safety signals) have been shown to develop powerful conditioned reinforcing properties, to support AA behavior through a habit-like mechanism (Dinsmoor and Sears, 1973; Morris, 1975; Fernando et al., 2014). In the present study, we found that rats continued to emit the lever press response and run faster in trials in which the warning cue (signaling the aversive event) was presented, as opposed to those in which the warning cue was not presented. Importantly, the safety signal continued to be presented in both the cued and non-cued trials, thus, if animals were actively avoiding for safety, then they would have continued to lever press at comparable levels in the cued and non-cued trials. This was not what we observed. Together with further evidence that the presentation of the safety signal alone did not promote lever pressing, we are confident that the motivation to actively avoid in the present study was largely sustained by the warning cue (negative reinforcement), rather than the elicitation of the safety cue (positive reinforcement), or any other events such as the insertion of the lever or removal of the guillotine door from the start compartment.

### Repeated Cocaine Exposure and the Acquisition of the AA Response

It is important to note that we did not observe any alterations in the acquisition of the conditioned AA response as a result of repeated cocaine exposure in the present study. This is consistent with the findings of previous investigations on the effects of psychostimulant drug exposure on conditioned AA (Riley and Foss, 1991; Murphy et al., 2001). Rat pups of mothers who were chronically exposed to cocaine during pregnancy showed normal acquisition of conditioned AA when training occurred during adulthood (Riley and Foss, 1991). Rats pre-exposed to cocaine or AMPH also showed normal acquisition of conditioned AA, although acquisition was impaired if the rats were pre-exposed to the conditioned stimulus prior to conditioned AA training (Murphy et al., 2001). Others have shown that when systemically injected with cocaine or AMPH immediately after conditioned escape training, rats and mice show enhanced AA when tested the following day (Janak and Martinez, 1992; Janak et al., 1992; Weinberger et al., 1992).

### Implications for Human Drug Addiction

Finally, the present findings suggest that repeated cocaine pre-exposure may lead to an imbalance in basic positive and negative motivational drives that could increase the susceptibility of addicts continuing drug-seeking and use despite harmful consequences. However, we acknowledge the fact that drug addiction is a multifaceted disorder with a number of contributing factors besides deficits in motivational processes (Deroche-gamonet et al., 2004). Cocaine pre-exposure has widespread effects on cortical and corticostriatal neurotransmission that are likely to lead to deficits in associative learning, and a reduction in inhibitory control over behavior (Everitt and Robbins, 2005; Goldstein and Volkow, 2011; Hearing et al., 2013; Volkow and Baler, 2014). Furthermore, given that only a small portion of human cocaine users develops addiction-like behaviors, it cannot be assumed that behavioral sensitization alone is representative of the addiction process. Indeed, studies that claim to have more closely modeled addiction-like behaviors in animals have shown that animals exhibit escalated drug self-administration if they are given long duration access to the drug but not if they are given short duration access to the drug, and that only short duration access is linked to locomotor sensitization while long duration access is not (Ben-Shahar et al., 2004; Lenoir and Ahmed, 2007). It is possible that the neurochemical and behavioral changes that occur following the completion of a short-term drug sensitization regimen (7 days pre-exposure in the present study) are a reflection of drug-induced alterations that contribute to the development of addiction in the early stages of drug use, and not necessarily to long term addiction. The interpretation of results from non-contingent drug sensitization regimens in relation to addiction is further complicated by findings which suggest that animals that receive cocaine contingently in self-administration paradigms do not develop neurochemical and behavioral sensitization as readily as animals that have received cocaine non-contingently (Lecca et al., 2007).

## Conclusion

In the present study, we introduced a novel AA paradigm designed to assess negative incentive motivation, defined here as the degree to which the animal was motivated to avoid a cued foot shock outcome. With this paradigm, we demonstrated that cocaine pre-exposure attenuated negative incentive motivation, which suggests that previous cocaine exposure reduces the motivational drive to prevent the occurrence of aversive events. This effect may contribute to the addiction process by enabling appetitive drug seeking motivation to gain powerful control over behavior and promote drug seeking despite the persistent occurrence of negative consequences. Given our finding of a drug-induced attenuation in negative incentive motivation, along with a plethora of evidence from previous studies that have demonstrated drug-induced enhancements in positive incentive motivation (Lett, 1989; Horger et al., 1990, 1992; Mendrek et al., 1998; Deroche et al., 1999; Fiorino and Phillips, 1999a,b; Harmer and Phillips, 1999a; Wyvell and Berridge, 2001), we propose that a drug-induced bidirectional shift in incentive motivation, whereby reward seeking is enhanced while the motivation to avoid aversive events is diminished, may critically contribute to the development of addiction-like behaviors.

## Author Contributions

DN contributed to the design, data collection, data analysis and writing. YN in the data collection. SE in the design and writing. RI in the conception, design, data analysis and writing.

## Conflict of Interest Statement

The authors declare that the research was conducted in the absence of any commercial or financial relationships that could be construed as a potential conflict of interest. The reviewer CC and handling Editor declared their shared affiliation.
